# A Study of Dip-Coatable, High-Capacitance Ion Gel Dielectrics for 3D EWOD Device Fabrication

**DOI:** 10.3390/ma10010041

**Published:** 2017-01-05

**Authors:** Carlos E. Clement, Dongyue Jiang, Si Kuan Thio, Sung-Yong Park

**Affiliations:** Department of Mechanical Engineering, National University of Singapore, Block EA, #07-08, 9 Engineering Drive 1, Singapore 117576, Singapore; mpecec@nus.edu.sg (C.E.C.); mpejd@nus.edu.sg (D.J.); sikuan@u.nus.edu (S.K.T.)

**Keywords:** electrowetting-on-dielectric, dip-coating, ion gels, liquid prism, high capacitance

## Abstract

We present a dip-coatable, high-capacitance ion gel dielectric for scalable fabrication of three-dimensional (3D) electrowetting-on-dielectric (EWOD) devices such as an *n* × *n* liquid prism array. Due to the formation of a nanometer-thick electric double layer (EDL) capacitor, an ion gel dielectric offers two to three orders higher specific capacitance (*c* ≈ 10 μF/cm^2^) than that of conventional dielectrics such as SiO_2_. However, the previous spin-coating method used for gel layer deposition poses several issues for 3D EWOD device fabrication, particularly when assembling multiple modules. Not only does the spin-coating process require multiple repetitions per module, but the ion gel layer also comes in risks of damage or contamination due to handling errors caused during assembly. In addition, it was observed that the chemical formulation previously used for the spin-coating method causes the surface defects on the dip-coated gel layers and thus leads to poor EWOD performance. In this paper, we alternatively propose a dip-coating method with modified gel solutions to obtain defect-free, functional ion gel layers without the issues arising from the spin-coating method for 3D device fabrication. A dip-coating approach offers a single-step coating solution with the benefits of simplicity, scalability, and high throughput for deposition of high-capacitance gel layers on non-planar EWOD devices. An ion gel solution was prepared by combining the [EMIM][TFSI] ionic liquid and the [P(VDF-HFP)] copolymer at various wt % ratios in acetone solvent. Experimental studies were conducted to fully understand the effects of chemical composition ratios in the gel solution and how varying thicknesses of ion gel and Teflon layers affects EWOD performance. The effectiveness and potentiality of dip-coatable gel layers for 3D EWOD devices have been demonstrated through fabricating 5 × 1 arrayed liquid prisms using a single-step dip-coating method. Each prism module has been individually controlled to achieve spatial beam steering without the need for bulky mechanical moving parts.

## 1. Ion Gel Dielectrics for EWOD

Electrowetting-on-dielectric (EWOD) is an emerging small-scale liquid handling technology [1,2]. With an electric potential applied between a liquid droplet and a solid electrode, the charge redistribution modifies the surface energy and thus decreases the contact angle on the solid surface [3,4,5]. The Young-Lippmann equation mathematically describes the relationship between the applied voltage (*V*) and the resultant contact angle (*θ*) as [6]:

(1) $$\cos\theta =\cos\theta_{0}+\frac{1}{2\gamma }cV^{2}$$

where *θ*_0_ represents the initial contact angle of the droplet at zero potential, *γ* is the interfacial tension between two immiscible liquids, and *c* is the capacitance per unit area.

For many practical EWOD applications, the use of high-capacitance dielectrics is critical to induce large surface tension modulation at a given voltage input. To meet this requirement, various dielectrics such as silicon dioxide (SiO_2_), aluminum oxide (Al_2_O_3_), and tantalum pentoxide (Ta_2_O_5_) have been used [7,8,9,10]. However, these dielectric materials are fabricated by conventional integrated circuit (IC) processes such as plasma enhanced chemical vapor deposition (PECVD), atomic layer deposition (ALD) and sputtering; they are typically time-consuming and require complex and expensive laboratory setups such as high vacuum facilities. To avoid the need of such IC processes, polymer-based dielectric materials such as PDMS and SU-8 have been alternatively used via a low-cost, spin-coating method [11,12,13]. Nonetheless, not only do these materials provide a lower permittivity than that of SiO_2_, but using spin coating method would produce a thick dielectric layer in the order of tens or hundreds of micrometers. Consequently, a dielectric layer with such thickness and low permittivity would result in a very low capacitance and thus poor EWOD performance. To overcome these issues arising from conventional dielectrics commonly used for EWOD applications, our group recently reported an ion gel dielectric that provides two to three orders higher specific capacitance (*c* ≈ 10 μF/cm^2^) than that of conventional dielectrics, while being simply fabricated by a spin-coating method [4]. Such a high capacitance of the ion gel results from the nanometer-thick electric double layer (EDL) capacitor formed by free counter-ions compactly accumulated at the interface [14,15,16,17]. With the spin-coated gel layers, we had demonstrated full contact angle modulation at a lower voltage than that of conventional dielectrics such as SiO_2_ and Al_2_O_3_ [4]. Despite of our study on ion gel dielectrics showing low-voltage EWOD performance, the spin-coating method was not suited for 3D devices with non-planar structures. The following section discusses the problems of the previous spin-coating methodology, particularly for 3D EWOD applications such as *n* × *n* arrayed liquid prisms.

## 2. Ion Gel Layer Coating for 3D EWOD Device Fabrication

**Figure 1:**
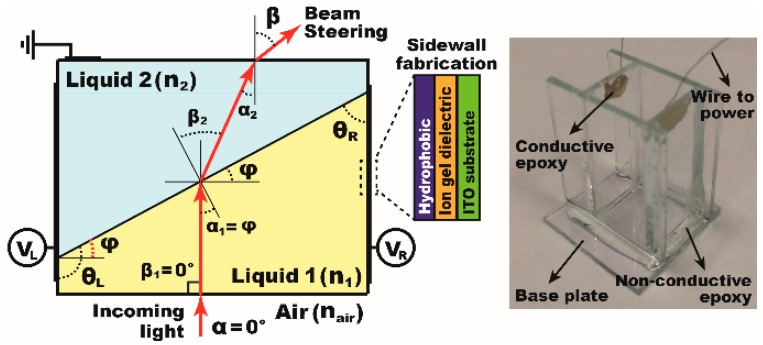
A rectangular prism is fabricated by assembling four conductive sidewalls coated with dielectric and hydrophobic layers and filled with two immiscible liquids. The electrowetting effect is used to adjust the prism apex angle *ϕ* by applying bias voltages (V_L_ and V_R_) to the left and right sidewalls, separately. Beam steering of the incoming light can be achieved at the interface of two media whose refractive indices are different (*n*_air_ ≠ *n*_1_ ≠ *n*_2_). The left figure shows the assembled prism device with a 10 × 10 mm^2^ aperture area before two liquids are placed.

With technical advances in EWOD over the years, recent attention has been extended to 3D devices, as they provide more flexibility and functionality than conventional 2D planar ones. For example, Fan et al. reported a wearable wristband assembled by four flexible EWOD modules where droplets were spatially manipulated on a curved wristband surface [12]. This lab-on-a-wristband (LOW) concept highlights the potential of 3D EWOD technology for point-of-care (POC) applications. Another example of the 3D EWOD devices popularly studied in recent years is the liquid prism that enables spatial beam steering [18,19,20,21,22,23]. EWOD-driven liquid prism technology has been used in diverse applications including sunlight beam steering for solar energy harvesting [24,25,26] and optical beam control to achieve a Fresnel lens [23]. By using fluids for light control, one advantage is the smooth interface of fluids formed due to the liquid’s tendency to minimize its surface energy. Such optical-grade smoothness of fluidic interfaces is very useful and cost-effective as it eliminates the need of high-precision fabrication or polishing processes typically required for solid optics [27]. In addition, the shape and position of liquid interfaces can be actively controlled using EWOD without bulky and complex mechanical moving parts [28]. This feature offers more reconfigurable prism devices for controlling their optical performances. A prism is fabricated by assembling four conductive sidewalls coated with a hydrophobic and a dielectric layer (Figure 1). Two immiscible liquids are filled up and enclosed by top and bottom transparent plates. Bias voltages (V_L_ and V_R_) are subsequently applied to the left and right sidewalls to induce the electrowetting effect to essentially control the prism apex angle (*ϕ*) [19,21,22]. In several previous studies, beam steering performance achieved by a single prism has been well described as a function of the refractive indices (*n*_1_ ≠ *n*_2_) of each medium as well as the apex angle (*ϕ*) [22,23,24]. Our group recently achieved the highest beam steering angle ever demonstrated up to *β* = 19.06° using a double-stacked prism configuration [22]. However, the aperture area is typically limited in a single prism due to surface tension modulation. This can be addressed by proposing an *n* × *n* arrayed prisms, which will be advantageous for solar energy applications [24]. Single prism modules can be joined to form an array, effectively increasing the overall aperture area where a large number of solar beams can be steered and focused onto a solar receiver with high concentrations reaching up to 2032× [24].

**Figure 2:**
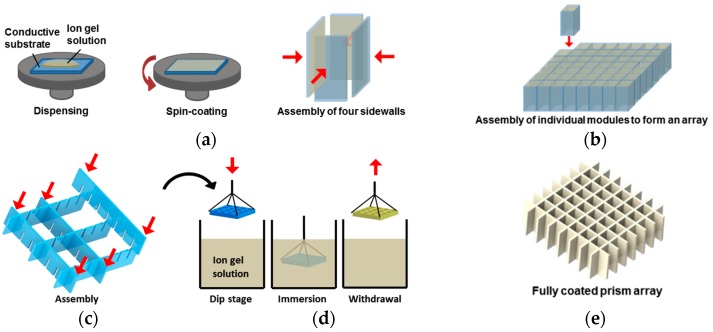
Schematics of typical spin- and dip-coating processes for fabricating arrayed liquid prisms as an example of 3D electrowetting-on-dielectric (EWOD) devices. (**a**) Four sidewall modules are separately spin-coated for gel layer deposition and then assembled to form a hollow 3D structure before being filled with two immiscible liquids; (**b**) For fabrication of *n* × *n* prism arrays, the spin-coating process needs to be repeated four times per module, i.e., 4*n*^2^, with the gel layers more prone to damage during assembly; (**c**) A dip-coating process completes a single-step coating of the gel layer over the entire surfaces of the 3D assembled structures. For example, substrates with finger-like protrusions are first assembled to form the arrayed 3D structure; (**d**) The whole assembly is then dip-coated for the gel layer coating on the entire surface area; (**e**) A dip-coating approach offers a single-step solution with the benefits of simplicity, scalability, and high throughput for deposition of high-capacitance gel layers on 3D EWOD devices, while fully eliminating the risk of layer damage or contamination that may occur during the assembling processes.

While the fabrication of such arrayed EWOD devices typically requires the assembly of multiple modules to form 3D structures, the previous spin-coated method poses several issues. Figure 2a illustrates the spin-coating procedure used to fabricate a single prism as an example of 3D EWOD devices coated with the gel layers. Considering that only a single substrate can be spin-coated at a time, the gel coating process has to be individually repeated for each of the four sidewalls. Next, they are carefully assembled into a hollow rectangular structure with four sidewalls whereby only the inner surfaces are coated with gel layers. However, during their assembly, the gel layer is at risk of damage or contamination from handling errors, which is detrimental to device performance and reliability. While such issues are already present for a single prism module, they are further compounded when fabricating multiple modules such as the case for *n* × *n* prism arrays (Figure 2b). The spin-coating step must be repeated four times per module, i.e., 4*n*^2^, and the more complicated assembling procedure increases the likelihood of ion gel damages. In addition, the chemical formulation (i.e., high concentrations of ionic liquids in gel solutions) previously used for a spin-coating method [4] causes the surface defects for the thin dip-coated layers, which led to poor EWOD performance. To obtain defect-free, robust gel layers without the issues arising from the spin-coating method aforementioned, we propose a dip-coating approach that not only offers a single-step coating solution to cover the gel layer over the entire surfaces of 3D structures, but also fully eliminates the risk of gel layer damage or contamination brought about during assembly. For the arrayed prism fabrication, substrates with finger-like protrusions are first assembled to form the hollow *n* × *n* arrayed structure as shown in Figure 2c. The whole assembly is then dip-coated to achieve the gel layer coating across the entire exposed surface area without requiring any additional steps as compared to the spin-coating method (Figure 2d,e).

In this paper, a dip-coatable high-capacitance ion gel dielectric is presented for scalable fabrication of 3D EWOD devices. We first investigated the effect of the chemical composition in gel solutions on EWOD performance. It was found that the high concentration of ionic liquids, which was typically used for the previous spin-coating method, causes the surface defects on the dip-coated gel layers. Large contents of copolymers (or a small amount of ion liquids) in gel solutions helps to remove the surface defects by increasing the melting point of the gel layers over the curing temperature of Teflon at 110 °C. Static EWOD experiments based on the improved fabrication process of employing dip-coating method together with a new chemical formulation were further conducted to understand the thicknesses effect of ion gel and Teflon layers on EWOD performance. The effectiveness of such dip-coatable gel layers for 3D device fabrication was demonstrated using 5 × 1 arrayed prisms with a 50 × 7 mm^2^ aperture area. A dip-coating approach offers a single-step solution with the benefits of simplicity, scalability, and high throughput for deposition of high-capacitance gel layers on 3D EWOD devices.

## 3. Materials and Methods

For the dip-coatable ion gel studies, we selected the [EMIM][TFSI] ionic liquid (1-ethyl-3-methylimidazolium bis(trifluoromethylsulfonyl)imide) and the [P(VDF-HFP)] copolymer (poly(vinylidene fluoride-co-hexafluoropropylene)), which are the same materials as the ones used in the previous spin-coating studies [4,14,15]. To prepare the ion gel solution, ionic liquid and copolymer were mixed at different weight ratios and further diluted in acetone to vary the solution viscosity, which is one of the variables to determine the thicknesses of the dip-coated gel layer [29]. For static EWOD studies, indium tin oxide (ITO)-coated substrates were dip-coated at various withdrawal speeds ranging from 100 to 800 mm/min, after which the samples were left to dry at room temperature for 24 h and cured at 70 °C for 4 h to purge all remaining acetone solvent. After full curing of the ion gel layer, the second hydrophobic layer was subsequently coated using a 6% Teflon AF solution (DuPont) through a dip-coating method at various withdrawal speeds and cured at 110 °C for 16 h. The thickness of both ion gel and Teflon layers were characterized using an optical profiler (ZYGO NewView 5032).

**Figure 3:**
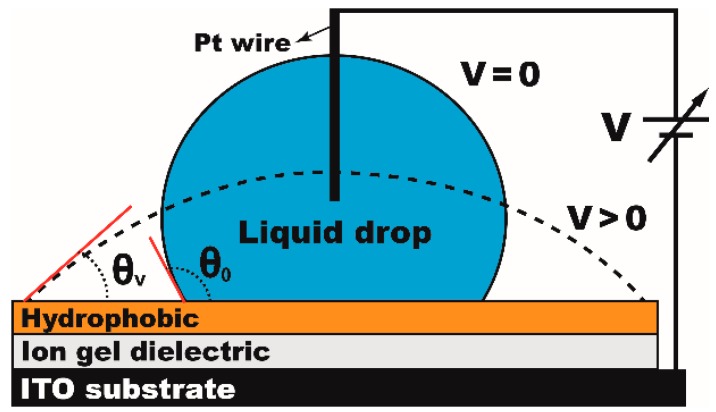
Experimental setup for static EWOD tests. A water droplet is placed on the ITO substrate dip-coated with the dielectric (ion gel) and hydrophobic (Teflon AF) layers.

Figure 3 shows the experimental setup for our static EWOD studies. A 7 μL water droplet was placed on the ITO substrate dip-coated with the ion gel and Teflon layers. A platinum (Pt) probe was then connected to the droplet as the ground electrode. The droplet contact angles were measured as the voltage input was increased in 5 V increments to understand the effects of chemical concentrations in the gel solution and thickness of each layer on EWOD performance.

## 4. Experimental Results

Since an ion gel layer is fabricated by combining an ionic liquid (IL) and a structuring copolymer (CP), it presents the features of both constituents such as the high capacitance contributed by the IL and the gel-like mechanical structure from the CP. Hence, the mixing ratio between two constituents has an important influence on the characteristics of the gel layer fabricated. For applications typically used for gating transistors, thin-film gel layers are fabricated by spin-coating gel solutions which are prepared at high concentrations of the IL ranging from 4:1 to 13:1 weight ratios with the CP [14,30,31]. High concentrations of the IL are critical to obtaining the ion gel layers with high ionic conductivity and thus faster switching speeds needed for gating transistors. Meanwhile, only a small amount of the CP would be sufficient to achieve gelation for mechanical integrity [15,32,33].

For our dip-coatable ion gel studies, we initially followed the previous spin-coating method [4,14,15,34] to prepare the ion gel solution, which included a high concentration of the IL at a 4:1 weight ratio with the CP. The gel solution was then dip-coated on an ITO substrate and cured at 70 °C, followed by the Teflon layer at 110 °C, respectively. Static EWOD tests were conducted to evaluate EWOD performance of the dip-coated gel layer. However, we soon realized that the previous spin-coating method including high concentrations of the IL in the gel solutions showed poor EWOD performance with the dip-coated gel layers. Bubbles generated by electrolysis had been consistently observed without any appreciable contact angle change. In the following sections, we discuss how the chemical formulation of the ion gel solution is key to obtaining robust, functional dip-coated gel layers. Using the gel solutions newly prepared, the thickness effects of both ion gel and Teflon layers were investigated on EWOD performance. Finally, the viability of the dip-coatable gel layers for 3D device fabrication was demonstrated by showing the 5 × 1 prism arrays whose apex angles were individually controlled.

### 4.1. Manifestation of Surface Defects on the Ion Gel Layer Dip-Coated

**Figure 4:**
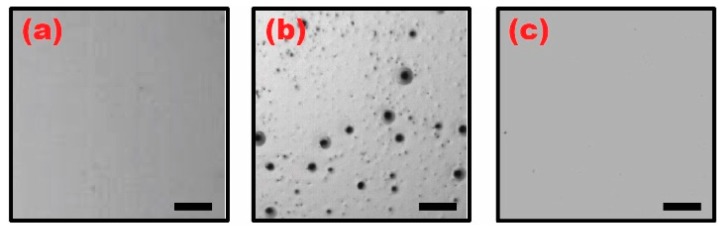
Microscopic images of the three different dip-coated samples. (**a**) An ion gel layer was dip-coated with the gel solution prepared with a high concentration of the ionic liquid (IL) at a 4:1 weight ratio with the copolymer (CP) and cured at 70 °C. A uniform surface quality is observed; (**b**) A Teflon layer is subsequently dip-coated over the gel layer and cured at 110 °C. Serious surface defects are observed; (**c**) For comparison, a pure Teflon layer was separately dip-coated and cured at 110 °C. No surface defects are observed. The scale bar represents a 0.5 mm length.

To understand how such poor EWOD performance occurred, we inspected the surface quality of the three different samples dip-coated with (a) a pure ion gel layer cured at 70 °C; (b) an ion gel layer baked at 70 °C over which an additional Teflon layer was cured at 110 °C; and (c) a pure Teflon layer baked at 110 °C. Figure 4 shows the microscopic images of these three samples. The ion gel layer was first dip-coated using a gel solution prepared with a high concentration of IL at a 4:1 weight ratio with the CP and cured at 70 °C. A uniform surface quality is observed for the pure ion gel layer (Figure 4a). However, when a Teflon layer was subsequently dip-coated over the gel layer and cured at 110 °C, abundant surface defects were clearly observed (Figure 4b). This was then compared to the case of a pure Teflon layer dip-coated and baked at 110 °C, where no surface defects were observed (Figure 4c). Based on our observations shown in Figure 4, it becomes evident that the surface defects manifested on the dip-coated gel layer only after the Teflon was dip-coated over it and cured at 110 °C, resulting in poor EWOD performance. Such a surface degradation phenomenon of the ion gel layer can be further reinforced by several previous studies. Jansen et al. experimentally showed that the melting point of the ion gel layer is significantly lowered when increasing the concentration of the [EMIM][TFSI] ionic liquid in the gel solution [35]. They observed that the neat [P(VDF-HFP)] copolymer has a melting point around 140 °C, which consequently drops down to 90 °C when the gel layer is made at a high weight ratio of 4:1 between the IL and the CP. This is because large contents of the CP (or a small amount of the IL) in the gel layer increases the crystallinity of the gelated layer and such large crystalline regions require more heat to melt than the amorphous regions [35]. In contrast, the solutions made with highly concentrated IL create less crystalline regions in the gel layer, resulting in an observed melting point depression. It was also reported that, when thin-film polymer layers are treated at a temperature higher than their melting points, the surface tension variations caused by phase separation induce surface deformation and roughness within the thin films [36]. In our experiments using high concentration of the IL at a 4:1 weight ratio with the CP, the ion gel layer underneath the Teflon layer experienced melting and migration during the Teflon curing period at 110 °C, which is above the 90 °C melting point of the gel layer [35,36]. This led to the formation of tiny aggregations, causing the surface defects as shown in Figure 4b. Hence, it was critical to understand how IL concentrations influence on EWOD performance of the dip-coated gel layers. Such is discussed in the next section.

### 4.2. Effect of Ionic Liquid Concentrations on EWOD

**Figure 5:**
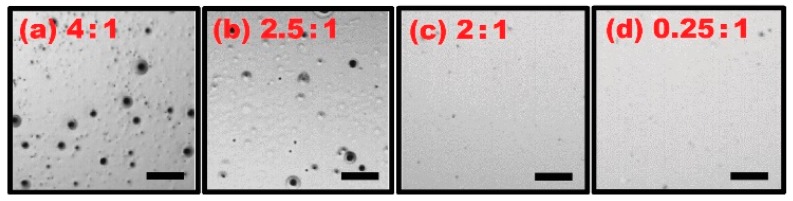
Microscopic images of the samples fabricated with various mixing ratios between the IL and the CP in the gel solution. The gel layers were dip-coated with the solutions made of the IL and the CP at various weight ratios of (**a**) 4:1; (**b**) 2.5:1; (**c**) 2:1; and (**d**) 0.25:1 and cured at 70 °C. The Teflon layer was subsequently dip-coated and baked at 110 °C. It is clearly observed that the surface defects decrease as the IL content is lowered relative to the CP in the mixture solution. The surface quality of the ion gel layer at the 2:1 and 0.25:1 weight ratios is greatly improved. The same solution concentration with respect to acetone was used for all the cases. The figure (**a**) is reprinted from Figure 3b. The scale bar represents a 0.5 mm length.

**Figure 6:**
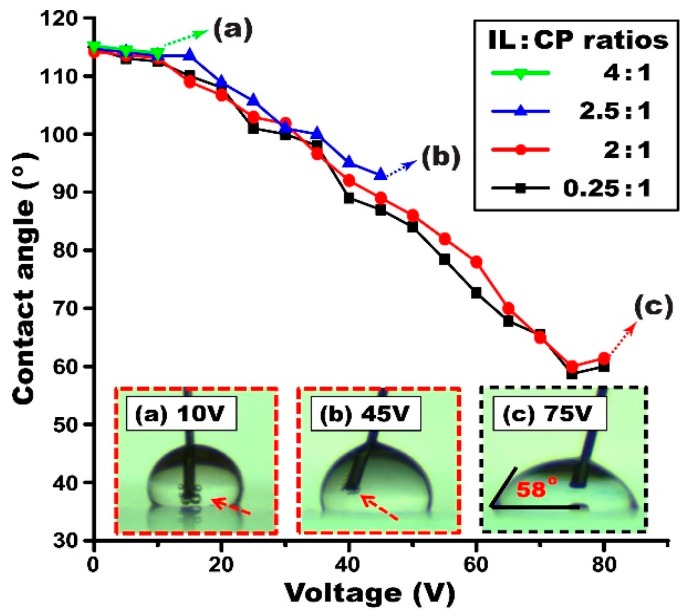
Effect of the IL concentrations in the gel solution on EWOD performance. The initial contact angle was similarly measured to be 115° on the Teflon surface for all the cases. EWOD performance was studied by measuring the contact angles with varying input voltages. Full contact angle modulation without electrolysis was achieved for the ion gel solutions prepared with low IL contents at 2:1 and 0.25:1 weight ratios with the CP. The other two cases at 4:1 and 2.5:1 showed poor EWOD performance. The insets (**a**,**b**) show the onset of bubbles generated by electrolysis (indicated by red arrows); while the inset (**c**) shows full contact angle modulation up to 58° achieved without electrolysis. All samples were prepared in the same experimental conditions, such as constant solution concentration, withdrawal speed, and a Teflon layer of 670 nm thickness.

For this study, several ion gel solutions were prepared by varying the weight ratios from 4:1 to 0.25:1 between the IL and the CP. These mixtures were further diluted in the same amount of acetone at 9%. Dip-coating and curing processes were implemented for both ion gel and Teflon layers, respectively. We inspected the surface quality of the samples dip-coated with the gel solutions prepared at various weight ratios and their microscopic images are shown in Figure 5. As discussed above, the surface defects were evident for the gel layer dip-coated with the solution containing a high concentration of the IL at a 4:1 weight ratio with the CP (Figure 5a). Such surface defects decrease, as the IL content is lowered relative to the CP (e.g., the layer fabricated at a 2.5:1 weight ratio shown in Figure 5b). For the solutions with low IL concentrations at 2:1 (Figure 5c) and 0.25:1 (Figure 5d), the surface defects are no longer observed. This is due to the effective increase of the gel layer’s melting point over the Teflon baking temperature at 110 °C as IL concentrations are lowered beyond the 2:1 ratio [35]. This results in the improvement of surface quality, as surface defects no longer arise from gel layer phase change. These samples shown in Figure 5 were subsequently tested for static EWOD experiments to examine the effect of the IL concentrations on EWOD performance. The graphs in Figure 6 present the droplet contact angles measured on the samples fabricated with four different weight ratios between the IL and the CP. As expected from the results in Figure 5, the ion gel samples fabricated at high IL weight ratios of 4:1 and 2.5:1 with the CP provided poor EWOD performance where bubbles were caused by electrolysis as a sign of dielectric failure (see Figure 6a,b). For the other cases with the low IL contents (e.g., 2:1 and 0.25:1 weight ratios), full contact angle modulation was achieved without electrolysis until reaching the contact angle saturation around 58° (see Figure 6c). From the experimental results in Figure 6, it is understood that the gel solution where the IL content is at least lower than the 2:1 weight ratio with the CP is required to obtain stable and robust dip-coated ion gel layers that enable full contact angle modulation without electrolysis. For all following experiments, samples were prepared using solutions with a low IL:CP ratio of 0.25:1.

### 4.3. Thickness Control of the Dip-Coated Gel Layers

**Figure 7:**
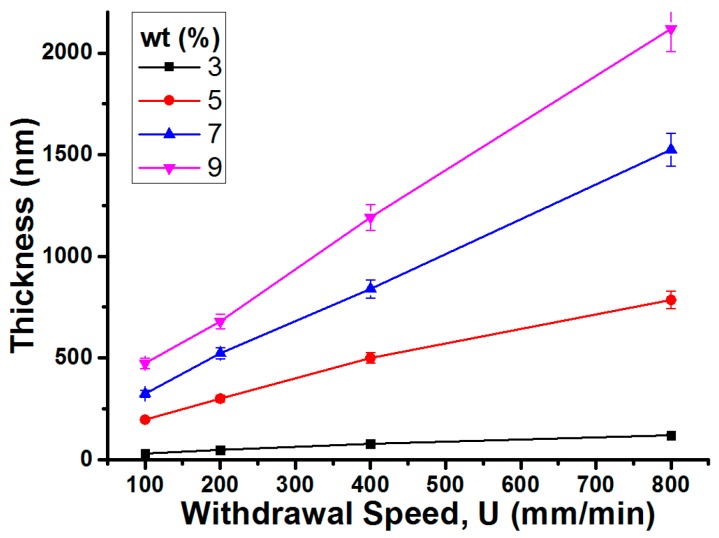
By adjusting the acetone concentrations and withdrawal speeds, we were able to vary the thicknesses of the dip-coated gel layer from 40 nm to 2.21 µm, which are a few orders thinner than the ones fabricated by the spin-coating method. The weight ratio between the IL and the CP was fixed at 0.25:1, while the amounts of acetone and withdrawal speeds were varied to control the layer thickness. The legend indicates the concentrations of the IL and the CP with respect to the acetone solvent.

According to the Landau-Levich derivation [29], the thickness of dip-coated layers is mainly determined by solution viscosity and withdrawal speed. To control the gel layer thickness by dip-coating, the gel solution was prepared at the fixed 0.25:1 weight ratio between the IL and CP, while overall solution concentrations (i.e., both IL and CP) with respect to an acetone solvent and withdrawal speeds were varied. Figure 7 presents the measured thicknesses of the dip-coated layers. Gel solutions with higher concentrations of the IL and the CP (e.g., 9%) in an acetone solvent are more viscous, consequently forming thicker layers. Likewise, dip coating at faster withdrawal speeds gives the wet film layer less time to develop, thus depositing thicker layers. It is worth noting that, for the given mixture ratio at 0.25:1, the solution viscosity was significantly high even at 9% and close to solute saturation. For the solutions at concentrations higher than 9%, the dip-coated samples showed immediate gelation and highly uneven surfaces and hence poor EWOD performance with electrolysis. This indicates that there is a limit to the thickness of functional ion gel layers to be produced by the dip-coating method. By adjusting the acetone concentrations and withdrawal speeds, we were able to vary the thicknesses of the dip-coated gel layer from 40 nm to 2.21 µm, which are a few orders thinner than the ones fabricated by the previous spin-coating method [4,14,15,34].

### 4.4. Thickness Effect of the Dip-Coated Gel Layers

It is known that ion gel layers provide a high specific capacitance (*c* ≈ 10 μF/cm^2^) as a result of the nanometer-thick electric double layer (EDL) capacitor formed by free counter-ions of ionic liquids compactly accumulated at the interface with an applied electric field [16,17,34]. This indicates that the intermediate gel layer constructed by structuring copolymers does not contribute to its capacitance [4,14,32], which is hence thickness independent, unlike conventional dielectrics whose capacitance is inversely proportional to their thickness. This thickness independence on capacitance was only verified for the thick ion gel layers (in the range of a few tens of micrometers) spin-coated with the gel solutions including high concentrations of the IL [4,14,15,34]. However, the dip-coated gel layers in our study not only are fabricated with the modified gel solutions, but also typically produce a few orders thinner gel layers (in the ranges of sub-micrometers) than that of spin coating. Therefore, it is worthwhile for us to study the thickness effect on EWOD performance of the dip-coated gel layers that have a different fabrication technique as compared to the previous spin-coating approach.

**Figure 8:**
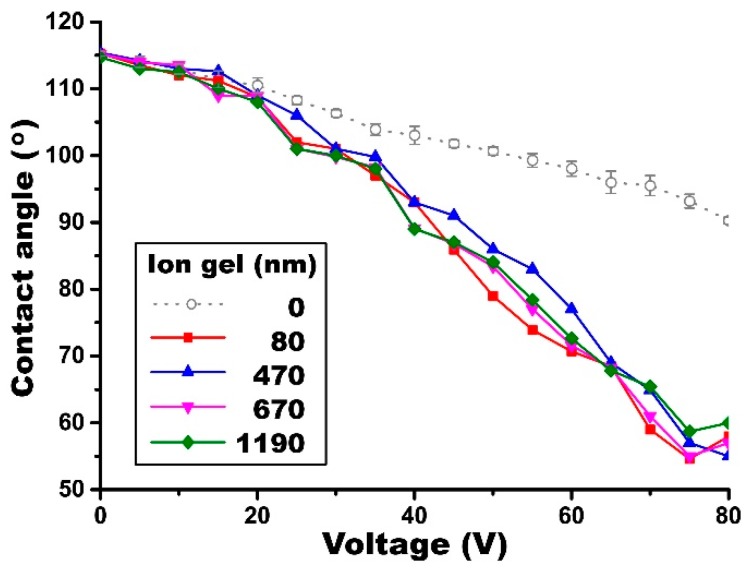
Thickness effect of the dip-coated gel layer on static EWOD performance. The gel layer thickness varies from 80 nm to 1.19 µm, while the Teflon layer was fixed at 670 nm for all experiments. The droplet contact angles were measured at various input voltages. A negligible difference in EWOD performance was observed for the gel layers of varying thicknesses. For comparison, data from a bare Teflon layer of the same thickness is added to highlight the significant contribution of the dip-coated gel layer.

For this study, an ion gel solution was prepared at the low concentration of the IL mixed with the CP at a 0.25:1 weight ratio. Then, the dip-coated gel samples were fabricated with various thicknesses ranging from 80 nm to 1.19 µm, while the Teflon layer was fixed at 670 nm atop of each gel layer. Static EWOD experiments were again conducted on these samples to see the thickness effect on the capacitance. Figure 8 shows the very similar contact angle changes regardless of the gel layer thicknesses. All the gel layers were capable of achieving full contact angle modulation without the practical issues of EWOD such as electrolysis and dielectric breakdown, with the contact angles saturating around 58° at 75 V. From the experimental results, the thickness independence of the ion gel’s capacitance holds true even for the thin dip-coated gel layers, which were fabricated with the gel solutions including low content of the IL. As a comparative study, a pure 670 nm thick Teflon layer over an ITO plate was tested and showed much poorer performance than that of the Teflon and ion gel stack, when it is used alone as a dielectric layer. Our study here demonstrated that a new dip-coating method using solutions with low IL concentrations (differently fabricated from the previous spin-coating method) is capable of producing defect-free, reliable dielectric layers as thin as 80 nm, thickness comparative with other conventional dielectrics commonly used for EWOD studies, by simply fabricating with a single-step dip-coating process.

### 4.5. Thickness Effect of the Dip-Coated Teflon Layers

**Figure 9:**
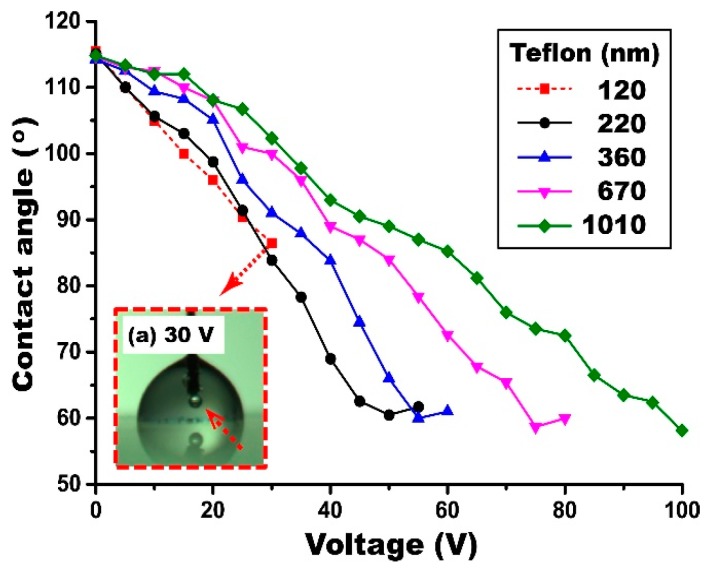
Thickness effect of the dip-coated Teflon layer on static EWOD performance. For all experiments, the gel layer was held fixed at 1.19 µm, while varying the Teflon layer thickness. A thin Teflon layer allows a high capacitance and thus the same angle modulation is achieved at a lower voltage than other thick layers. The inset shows bubble generation due to electrolysis when the Teflon layer is 120 nm thin at 30 V.

Since ion gel layers are fabricated by a new dip-coating method, it is also important to understand the thickness effect of the dip-coated Teflon layer on EWOD performance. With the gel layer fixed at 1.19 µm, the thickness of the Teflon layer was varied from 120 to 1010 nm. The contact angles were measured at varying voltage inputs and the results are shown in Figure 9. EWOD performance improved as decreasing the thickness of the Teflon layer. For example, full contact angle modulation was achieved around 60° at 50 V for the 220 nm thick Teflon layer, while the contact angle was around 92° for the 1010 nm thick layer at the same voltage input. These observations are very consistent with our general understanding of EWOD performance, i.e., the capacitance is inversely proportional to the layer thickness and thus a thin Teflon layer can achieve more contact angle modulation than that of a thick layer at the same bias voltage. However, for very thin Teflon layers such as the case of 120 nm, electrolysis takes place at 30 V before full contact angle modulation (see Figure 9a). Since the capacitance of the ion gel is a few orders higher than that of the Teflon, nearly 99% of the voltage input is distributed across the hydrophobic layer when the two capacitors of the ion gel and Teflon layers are connected in series, as shown in Figure 3. The theoretical breakdown voltage for the 120 nm thick Teflon layer was estimated as 24 V [9]. Nevertheless, the droplet contact angle was further modulated even at the voltage of 30 V exceeding this breakdown threshold. This observation can be explained by several previous studies [37,38], reporting that, although the voltage distributed across the Teflon layer exceeds its breakdown voltage, the device performs continuously while also slowly degrading.

### 4.6. Demonstration of the Dip-Coatable Ion Gel for an Arrayed Prism as an Example of 3D EWOD Devices

**Figure 10:**
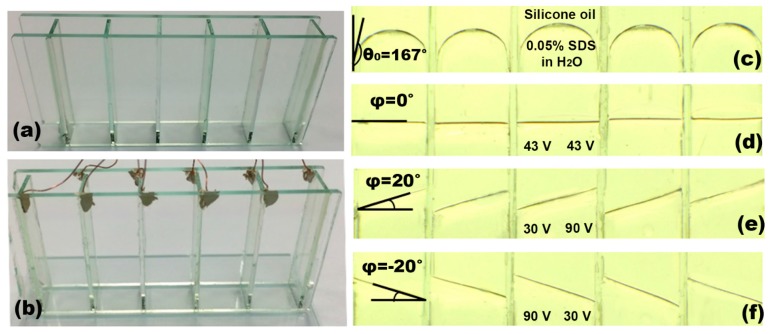
Demonstration of the dip-coatable ion gel fabrication for 5 × 1 arrayed liquid prisms as an example of 3D EWOD devices. (**a**) Five adjacent hollow structures were first assembled with multiple sidewalls modules; (**b**) The depositions of the ion gel and Teflon layers were completed through a single-step dip-coating method, which offers the benefits of simplicity, scalability, and high throughput over spin coating. The sidewalls were connected to a multi-channel power supply for individual voltage control, while a base substrate served as a ground electrode; (**c**) The arrayed prisms were filled with silicone oil and water added with a 0.05% SDS surfactant. The curved meniscus was initially formed with the contact angle of 167° at the interfaces between the two immiscible liquids; (**d**) When external bias voltages were equally applied to the sidewall (V_L_ = V_R_ = 43 V), the apex angles were set to *ϕ* = 0° and all five prisms were able to achieve a flat interface; (**e**) The interface was controlled with the apex angle of *ϕ* = 20° when V_L_ = 30 V and V_R_ = 90 V; (**f**) Similarly, the interface was titled to *ϕ* = −20° when V_L_ = 90 V and V_R_ = 30 V.

**Figure 11:**
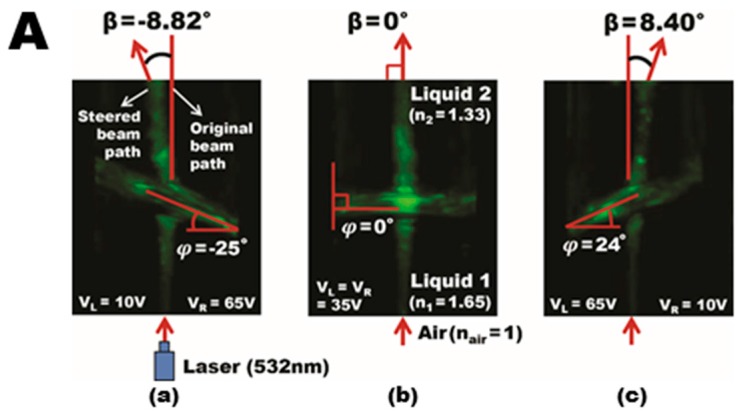

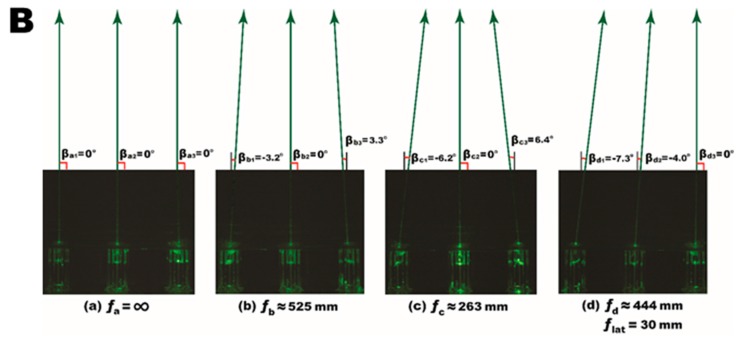
Experimental demonstrations of beam steering with the dip-coatable 3D liquid prisms. (**A**) The top images show the beam deflection using a single prism; (**a**) At *ϕ* = −25°, the beam steering was achieved as large as *β* = −8.82°; (**b**) At *ϕ* = 0°, a laser beam passes perpendicularly through the interface; (**c**) Similarly, we can achieve beam steering of *β* = 8.40° at *φ* = 24° (reprinted from the Reference [20] with the permission). (**B**) The bottom images present arrayed liquid prims that enable 3D focal tuning by controlling arrayed prisms; (**a**) When all steering angles are zero, *β*_a1_ = *β*_a2_ = *β*_a3_ = 0°, the Fresnel lens does not perform as a concentrating element (i.e., *f*_a_ = ∞); (**b**) When the outer prisms are modulated such that the steering is *β*_b1_ = −3.2°, *β*_b3_ = 3.3° and *β*_b2_ remains at 0°, the beams converge and the lens is then said to have a focal length at *ƒ*_b_ ≈ 525 mm; (**c**) Further manipulation at higher angles of *β*_c1_ = −6.2°, *β*_c2_ = 0°, and *β*_c3_ = 6.4° allows the focal point to reduce from *ƒ*_b_ ≈ 525 mm to *ƒ*_c_ ≈ 263 mm; (**d**) By controlling the arrayed prisms (*β*_d1_ = −7.3°, *β*_d2_ = −4.0°, and *β*_d3_ = 0°) non-symmetrically, spatial focal tuning has been achieved in both longitudinal and lateral directions at *ƒ*_d_ ≈ 444 mm and *ƒ*_lat_ = 30 mm (reprinted from [21] with the permission).

To demonstrate the viability of our dip-coated ion gel for 3D EWOD devices, a 5 × 1 liquid prism array were fabricated with an aperture area of 50 × 7 mm^2^ as an example of 3D devices. Multiple sidewalls were first assembled to form five adjacent hollow structures (Figure 10a). Copper wires were connected on each sidewall using a conductive adhesive. The gel solution was prepared at a 0.25:1 weight ratio between the [EMIM][TFSI] ionic liquid and the [P(VDF-HFP)] copolymer, which was further diluted in acetone at 3%. A single-step dip-coating procedure was conducted to deposit the ion gel dielectric layer over the arrayed 3D hollow structures, which offers the benefits of simplicity, scalability, and high throughput over the spin-coating method as discussed in Section 2. After curing the gel layer coated on the 3D device surfaces at 70 °C, another dip-coating and baking processes at 110 °C were carried out to provide a hydrophobic (Teflon) layer. Figure 10b presents the fully assembled 3D structure of the arrayed prisms where an additional ITO base plate serves as the common ground electrode. Two immiscible liquids, water and silicone oil, were then filled in each of the prisms. To improve interfacial stability, a 0.05 wt % sodium dodecyl sulfate (SDS) surfactant was added to water [39]. Due to Laplace pressure difference, its initial contact angle is measured at 167°, as illustrated in Figure 10c. For operation of the liquid prism array fabricated, we built a LabView-programmed multi-channel power supply (Keysight N6700B) which can separately control the bias voltages applied to all ten inner sidewalls to achieve individual control of the five prisms. Figure 10d–f show the versatility of our prism arrays that are formed with the apex angles, ranging −20° ≤ *ϕ* ≤ 20° by varying the applied voltages at each of the prism sidewalls. By using two liquids with different refractive indices (*n*_air_ ≠ *n*_1_ ≠ *n*_2_), the previous studies have demonstrated beam steering of the incoming light that can be effectively achieved without the need of bulky mechanical moving parts [22,23,24]. Figure 11a shows an experimental demonstration of beam steering using a single liquid prism filled with high-reflective-index oil 1-bromonapthalene (liquid 1) and water (liquid 2). Beam steering angles of *β* = −8.82°, 0°, 8.40° were achieved when voltages were applied due to respective prism apex angle modulations as a result of the large difference in refractive indices between the two liquids [22]. Our group recently achieved the highest beam steering angle ever demonstrated up to *β* = 19.06° using a double-stacked prism configuration [22]. Supplementary to the double-stacked prism configuration, we have experimentally demonstrated spatial focal tuning using 3 × 1 linearly arrayed liquid prisms, as illustrated in Figure 11b. Each prism in the array can be individually controlled to replicate the subsections of a conventional Fresnel lens. Incoming light can then be steered accordingly to vary the focal length of the lens along both the longitudinal and lateral directions, thus making *x*- and *z*-axis spatial tuning possible [23].

Experimental results demonstrate that the proposed dip-coating method effectively provides a single-step coating solution to provide defect-free, functional gel layers for 3D EWOD device fabrication, which is much simpler and more convenient than spin coating. Although we have shown arrayed prisms as examples of 3D EWOD devices, the advantages of our proposed method will become significantly more evident when fabricating devices with more complex geometries such as *n* × *n* arrayed prism panels and other non-planar structures.

## 5. Conclusions

This work presents a dip-coatable high-capacitance ion gel dielectric for 3D EWOD device fabrication that typically requires the assembly of multiple modules to form non-planar structures. A dip-coating approach offers a single-step solution with the benefits of simplicity, scalability, and high throughput for deposition of high-capacitance gel layers on 3D EWOD devices. With the dip-coated ion gel layers fabricated from an [EMIM][TFSI] ionic liquid and a [P(VDF-HFP)] copolymer, our experimental tests showed that the previous spin-coating method for preparation of the ion gel solution cannot be used for EWOD due to the surface defects that manifested when the gels were subject to the high temperatures needed for curing the hydrophobic (Teflon) layer. A new formulation lowering the content of ionic liquids in the gel solution was developed to obtain defect-free robust gel layers. The following experimental studies further showed the thickness effects of the ion gel and Teflon layers on EWOD performance. It was found to be highly dependent on the thickness of the hydrophobic layer, yet largely independent of the dip-coated gel thickness. Finally, we demonstrated the viability of our proposed dip-coating method using the 5 × 1 arrayed prisms as an example of 3D EWOD devices. With the dip-coated ion gels, our prism array was able to achieve individual prism control with the apex angle steering of up to ±20°.

The beauty of the proposed approach lies in providing reliable EWOD performance with simple and scalable processes. The single-step dip-coating process becomes greatly advantageous when fabricating 3D devices with more complex geometries such as *n* × *n* arrayed liquid prism panels and other non-planar structures. Our study here using the dip-coatable high-capacitance ion gel dielectric will have numerous potential uses in the growing field of 3D EWOD devices.
